# Mapping and Sequencing of a Significant Quantitative Trait Locus Affecting Resistance to Koi Herpesvirus in Common Carp

**DOI:** 10.1534/g3.118.200593

**Published:** 2018-08-27

**Authors:** Christos Palaiokostas, Diego Robledo, Tomas Vesely, Martin Prchal, Dagmar Pokorova, Veronika Piackova, Lubomir Pojezdal, Martin Kocour, Ross D. Houston

**Affiliations:** * The Roslin Institute, Royal (Dick) School of Veterinary Studies, University of Edinburgh, Easter Bush, Midlothian, EH25 9RG, Scotland, United Kingdom; † Department of Animal Breeding and Genetics, Swedish University of Agricultural Sciences, Box 7090, 750 07 Uppsala, Sweden; ‡ Veterinary Research Institute, Hudcova 70, Brno 62100, Czech Republic; § University of South Bohemia in Ceske Budejovice, Faculty of Fisheries and Protection of Waters, South Bohemian Research Centre of Aquaculture and Biodiversity of Hydrocenoses, Zatisi 728/II, 389 25 Vodnany, Czech Republic

**Keywords:** Carp, Koi herpes virus, RADseq, GWAS

## Abstract

Cyprinids are the most highly produced group of fishes globally, with common carp being one of the most valuable species of the group. Koi herpesvirus (KHV) infections can result in high levels of mortality, causing major economic losses, and is listed as a notifiable disease by the World Organization for Animal Health. Selective breeding for host resistance has the potential to reduce morbidity and losses due to KHV. Therefore, improving knowledge about host resistance and methods of incorporating genomic data into breeding for resistance may contribute to a decrease in economic losses in carp farming. In the current study, a population of 1,425 carp juveniles, originating from a factorial cross between 40 sires and 20 dams was challenged with KHV. Mortalities and survivors were recorded and sampled for genotyping by sequencing using Restriction Site-Associated DNA sequencing (RADseq). Genome-wide association analyses were performed to investigate the genetic architecture of resistance to KHV. A genome-wide significant QTL affecting resistance to KHV was identified on linkage group 44, explaining approximately 7% of the additive genetic variance. Pooled whole genome resequencing of a subset of resistant (n = 60) and susceptible animals (n = 60) was performed to characterize QTL regions, including identification of putative candidate genes and functional annotation of associated polymorphisms. The TRIM25 gene was identified as a promising positional and functional candidate within the QTL region of LG 44, and a putative premature stop mutation in this gene was discovered.

Common carp (*Cyprinus carpio* and *Cyprinus rubrofuscus*), is one of the most highly produced aquaculture fish species globally (FAO 2015), being farmed in a wide variety of environments and production systems (Balon 1995). However, in common with many aquaculture species, only a minority of farmed carp are derived from family-based selective breeding programs (Vandeputte 2003; Janssen *et al.* 2017). The potential for selective breeding to enhance production in carp is highlighted by several studies, but much of the production of commercial stock is still generated via intraspecific crossbreeding (Kocour *et al.* 2005, 2007; Vandeputte *et al.* 2008; Nielsen *et al.* 2010; Prchal *et al.* 2018).

Koi herpesvirus (KHV), also known as Cyprinid herpesvirus-3 (CyHV-3), is one of the main threats to carp production. The first major outbreaks were recorded in 1998 (Hedrick *et al.* 2000), and subsequent outbreaks in many carp producing countries were reported worldwide (Haenen *et al.* 2004). The seriousness of the KHV threat is highlighted by its listing as a notifiable disease by the European Union (Taylor *et al.* 2010) and the World Organization for Animal Health (OIE 2018). Selective breeding is a valuable tool for contributing to sustainable food production through the prevention and management of infectious outbreaks in a wide range of species (Bishop and Woolliams 2014). This may be particularly true in aquaculture species, due to moderate to high heritabilities of disease resistance documented in numerous cases (Ødegård *et al.* 2011; Houston 2017), and successful examples of disease control using marker-assisted breeding, *e.g.*, the case of the IPN virus in Atlantic salmon; *Salmo salar* (Houston *et al.* 2008; Moen *et al.* 2009).

Several studies have investigated the genetic basis of KHV resistance in carp (utilizing data and samples collected from disease challenge trials), showing encouraging results with large variation in survival both between families (Dixon *et al.* 2009; Tadmor-Levi *et al.* 2017) and between strains (Shapira *et al.* 2005; Piačková *et al.* 2013). Results from candidate gene association studies have suggested a possible role for polymorphism in MHC loci (Rakus *et al.* 2009) and Interleukin-11 (Kongchum *et al.* 2011) in host resistance to KHV. Taken together, these studies indicate that selective breeding has the potential to increase resistance to KHV, with potential downstream benefits for the carp aquaculture industry and fish welfare. However, to date, genome-wide polymorphisms have not been applied to investigate the genetic architecture of resistance to KHV.

Restriction-site associated DNA sequencing (RADseq) (Baird *et al.* 2008) and similar genotyping by sequencing techniques have been widely applied to generated genome-wide SNP markers due to their cost-efficiency in a wide range of aquaculture species (Robledo *et al.* 2017), including common carp ((Palaiokostas *et al.* 2018a). Various genome wide association studies (GWAS) using this technique have been published in aquaculture species (*e.g.*, Campbell *et al.* 2014; Palti *et al.* 2015). GWAS have been used to study disease resistance in various aquaculture species including salmonids (Correa *et al.* 2015, 2017; Vallejo *et al.* 2017; Barría *et al.* 2018; Robledo *et al.* 2018), catfish (Zhou *et al.* 2017), European sea bass (Palaiokostas *et al.* 2018b) and Pacific oyster (Gutierrez *et al.* 2018) among others. With the notable exception of the aforementioned case of IPN resistance in salmon, the GWAS results have pointed to a polygenic or oligogenic architecture for disease resistance in aquaculture species. The main aim of this study was to investigate genetic resistance to KHV in common carp using a RADseq approach. Classical genome wide association study (GWAS) and weighted genomic best linear unbiased predictor (WGBLUP) approaches were taken to examine the genetic architecture of resistance. Finally, pooled whole genome sequencing (PWGS) was performed in a subset of samples with divergent resistance and susceptibility to characterize and annotate QTL regions, and to identify potential gene candidates and polymorphisms involved in KHV resistance.

## Materials and Methods

### Sample collection and disease challenge

A population of Amur Mirror Carp was created at the University of South Bohemia in České Budějovice, Czech Republic in May 2014 using artificial insemination (Vandeputte *et al.* 2004) involving four factorial crosses of five dams x ten sires (20 dams and 40 sires in total). Incubation of eggs was performed in 9 L Zugar jars at 20°. At the first swimming stage, randomly sampled progeny from each mating (of approximately equal total volume) were pooled and stocked into several nursery earthen ponds at stocking density of 150,000 larvae / ha and reared under semi-intensive pond conditions throughout the growing season (from May to September). Before the challenge test a random sample of 1,500 fish described above were tagged and fin clipped for DNA extraction. These fish were the same as those described in Palaiokostas *et al.* (2018a). These animals were acclimatized for five days at water temperature of 22° and bathed in FMC solution (formalin, malachite green, methylene blue using a dose of 2 mL per 100 L of water) to eliminate ectoparasites. Subsequently, the fish were transferred to Veterinary Research Institute (VRI) in Brno (Czech Republic) to perform the KHV disease challenge test. A small (n = 215) sample of koi carp were challenged alongside the Amur mirror carp as a positive control, since Koi carp are highly susceptible to KHV.

A cohabitation challenge was performed in a 1,400 L tank equipped with recirculation and biological filtration. Koi carp received an intraperitoneal injection with 0.2 mL culture medium containing 10^4^ TCID 50 / mL KHV at day 0 and were added into the tank with challenged fish. Mortality of individual fish was recorded twice a day for a period of 35 days post infection (dpi). Presence of KHV on a sample of dead fish (n = 100) was confirmed by PCR according to guidelines by the Centre for Environment, Fisheries & Aquaculture Science, UK (Cefas) (Pokorova *et al.* 2010). The experiment was run until mortalities were negligible, implying that survivors were resistant. The entire experiment was conducted in accordance with the law on the protection of animals against cruelty (Act no. 246/1992 Coll. of the Czech Republic) upon its approval by Institutional Animal Care and Use Committee (IACUC) of the VRI and appropriate state authority. All people conducting the experiment hold a certificate about qualification to conduct experiments on the live animals, and the VRI is accredited for the culture of experimental animals according to the aforementioned law.

### Library preparation and sequencing

The RAD library preparation protocol followed the methodology originally described in Baird *et al.* (2008) and presently in detail in Palaiokostas *et al.* (2018a). Briefly, template DNA was digested using the *Sbf*I (recognizing the CCTGCA|GG motif) high fidelity restriction enzyme (New England Biolabs; NEB). DNA shearing was conducted with a Pico bioruptor (Diagenode). Following a final gel elution step into 20 µL EB buffer (MinElute Gel Purification Kit, Qiagen), 66 libraries (24 animals each) were sent to BMR Genomics (Italy), for quality control and high-throughput sequencing. RAD libraries were run in fourteen lanes of an Illumina NextSeq 500, using 75 base paired-end reads (v2 chemistry).

Whole genome sequencing libraries (n = 4) from pooled DNA samples (30 animals each library) of susceptible and resistant animals were constructed using the Illumina TruSeq DNA PCR free kit (350bp insert). Sequencing was performed in Edinburgh Genomics facilities using two lanes of Illumina HiSeq 4000.

### SNP discovery and genotyping

The process of obtaining the SNP genotype data from the RADseq reads was described in detail in Palaiokostas *et al.* (2018a). Briefly, sequenced reads were aligned to the common carp reference genome assembly version *GCA_000951615.2* (Xu *et al.* 2014) using bowtie2 (Langmead and Salzberg 2012). The aligned reads were sorted into RAD loci and SNPs were identified using the Stacks software 1.4 (Catchen *et al.* 2011). The SNPs were detected using a minimum stack depth of at least ten or five for the parental and offspring samples respectively. SNPs with minor allele frequency (MAF) below 0.01, greater than 20% missing data, and deviating from expected Hardy-Weinberg equilibrium in the parental samples (*P* < 1e-06) were discarded. R/hsphase (Ferdosi *et al.* 2014) software was used for parentage assignment allowing for a maximum genotyping error of 4%. The pedigree obtained was further validated for possible erroneous assignments using FImpute (Sargolzaei *et al.* 2014). In total, 1,214 offspring were uniquely assigned, forming 195 full-sib families (40 sires, 20 dams). Since the carp reference genome assembly is currently very fragemented, a medium density linkage map of 12,311 SNPs grouped in 50 linkage groups was created (Palaiokostas *et al.* 2018a), and used to orientate the results from the GWAS.

### Heritability estimation

The probit link function was used to connect the observed binary phenotype (0 = dead, 1 = alive) with the underlying liability scale. Variance components were estimated using the R/BGLR (Pérez and de Los Campos 2014) software with the following animal model:

l=Xb + Zu + e, (1)

where **l** is the vector of latent variables, **b** is the vector of the fixed effects (cross, standard length), **X** is the incidence matrix relating phenotypes with the fixed effects, **Z** is the incidence matrix relating phenotypes with the random animal effects, **u** is the vector of random animal effects ∼ N(0, **A**σ_g_^2^) [where **A** corresponds to the pedigree-based relationship matrix and is replaced by G for analyses using the genomic relationship matrix (VanRaden 2008) and σ_g_^2^ is the additive genetic variance], **e** the vector of residuals ∼N(0, **I$\sigma_{\text{e}}^{2}$**) where $\sigma_{e}^{2}$ is the residual variance.

The parameters of this model were estimated through Markov chain Monte Carlo (MCMC) using Gibbs sampling (11 M iterations; burn-in: 1 M; thin: 1,000). Convergence of the resulting posterior distributions was assessed both visually (inspecting the resulting MCMC plots) and analytically using R/coda v0.19-1 (Plummer *et al.* 2006). Heritability for the trait of survival during the KHV challenge (on the underlying liability scale) was estimated using the following formula:

$$\;h^{2}=\frac{\sigma_{\text{g}}^{2}}{\sigma_{\text{g}}^{2}+\sigma_{\text{e}}^{2}}\;$$

where $\sigma_{g}^{2}$ is the previous estimated additive genetic variance and $\sigma_{e}^{2}$ the residual variance. Residual variance on the underlying scale is not identifiable in threshold models (Goldstein *et al.* 2002; Nakagawa and Schielzeth 2013) and was therefore fixed to 1.

### Genome wide association analysis (GWAS)

To test the association between individual SNPs and resistance to KHV, a classical genome wide association study (CGWAS) was performed using R/gaston (Perdry and Dandine-Roulland 2016). The mixed model applied for overall survival had the same format as in (1) with the addition of including each SNP as a fixed effect. The variance components were estimated using the penalized quasi-likelihood approach (Chen *et al.* 2016). The genome-wide significance threshold was calculated using a Bonferroni correction (0.05 / N), where N represents the number of tested SNPs.

Weighted genomic best linear unbiased predictor (WGBLUP) was performed (Wang *et al.* 2012) using direct genomic values (DGV) (Lourenco *et al.* 2015; Zhang *et al.* 2016). The weighted genomic relationship matrix was initially created following VanRaden (2008) as:

$$G^{*}=\mathrm{ZD}Z^{\prime }q$$

where **Z** *is* the design matrix relating genotypes of each locus, **D** is a weight matrix for all SNPs, and **q** is a weighting vector derived from observed SNP frequencies. SNP weights were calculated using the nonlinearA method (VanRaden 2008). Briefly the steps for performing WGBLUP were as follows (Wang *et al.* 2012):

- Initialize **D** = **I** and t = 1, where **I** the identity matrix and t is the iteration number.
- Calculate G*.
- Estimate DGVs.
- Estimate SNP effects from GEBVs: **$\hat{\alpha }=\mathrm{qD}Z^{\prime }G^{*}\hat{u}$**, where $\hat{\alpha }$ the vector of SNP effects and $\hat{u}$ the vector of DGV
- Calculate the weight for each SNP: $d_{ii}^{\left(t+1\right)}=1.125^{\frac{\left|{\hat{a}}_{i}\right|}{sd(\hat{a})}-2\;}$, where ${\hat{a}}_{i}$ the estimated SNP effect (VanRaden 2008).
- Normalize SNP weights so the total genetic variance remains constant.
- Loop to step d) until convergence (10^−14^).

Convergence of SNP weights was tested using the convergence criterion BLUPF90 uses for variance components estimation

$$\text{C}=\frac{\underset{i}{\sum }{\left(\theta_{i}-\;\xi_{i}\right)}^{2}}{\underset{i}{\sum }\theta_{i}^{2}}$$

Percentage of additive genetic variance was estimated by non-overlapping windows of 10 adjacent SNPs as follows:

$$\frac{\text{Var}({\text{α}}_{\text{i}})}{\sigma_{\text{g}}^{2}}\times \;100\%=\frac{\text{Var}(\overset{\text{i}=10}{\underset{\text{i}}{\sum }}{\text{z}}_{\text{i}}\hat{{\text{α}}_{\text{i}}})}{\sigma_{g}^{2}}\times \;100\%$$

where var(a_i_) the additive genetic variance of the tested window of adjacent SNPs and $\sigma_{\text{g}}^{2}$ the total additive genetic variance. The weighted GBLUP analyses were performed using THRGIBBSF90 for estimating DGVs (Misztal *et al.* 2002, 2018) combined with iterations of PreGSF90 and PostGSF90 (Aguilar *et al.* 2011) until convergence (10^−14^).

### Pooled whole genome sequencing analysis

Pools of genomic DNA (25 ng / ul) from 60 survivors and 60 mortalities from the disease challenge experiment were prepared. These animals originated from 20 full-sib families, and the family structure was balanced between the resistant and susceptible pools. Libraries were prepared using the TruSeq DNA PCR free kit (350 bp insert size) and sequenced in two lanes of an Illumina HiSeq 4000 using paired-end sequencing by Edinburgh Genomics.

Reads were QC-filtered (phred score above 30) and trimmed to 140 bp long using Trimmomatic v0.36 (Bolger *et al.* 2014). Reads were aligned to the carp reference genome *GCA_000951615.2* (Xu *et al.* 2014) using bowtie2 (Langmead and Salzberg 2012). SNP identification was performed using Burrows-Wheeler Aligner v0.7.8 (BWA-mem, Li 2013). Pileup files describing the base-pair information at each genomic position were generated from the alignment files using the mpileup function of Samtools v1.6 (Li *et al.* 2009) requiring minimum mapping and base quality of 20. A Cochran-Mantel-Haenszel test was performed to test the significance of the allele frequency differences using Popoolation 2 v1.201 (Kofler *et al.* 2011). Only those genomic positions with at least 6 reads of the alternative allele across all pools and a maximum coverage of 50 reads and a minimum of 8 in all pools were considered SNPs. All QC-filtered SNPs were annotated using SNPeff (Cingolani *et al.* 2012).

### Data availability

Raw reads were deposited in the National Centre for Biotechnology Information (NCBI) repository under project ID PRJNA414021. Table S1 contains the phenotypic data. Table S2 contains the pedigree. Table S3 contains the genotypic data. Supplemental material available at Figshare: https://doi.org/10.25387/g3.6281561.

## Results

### Disease challenge

**Figure 1:**
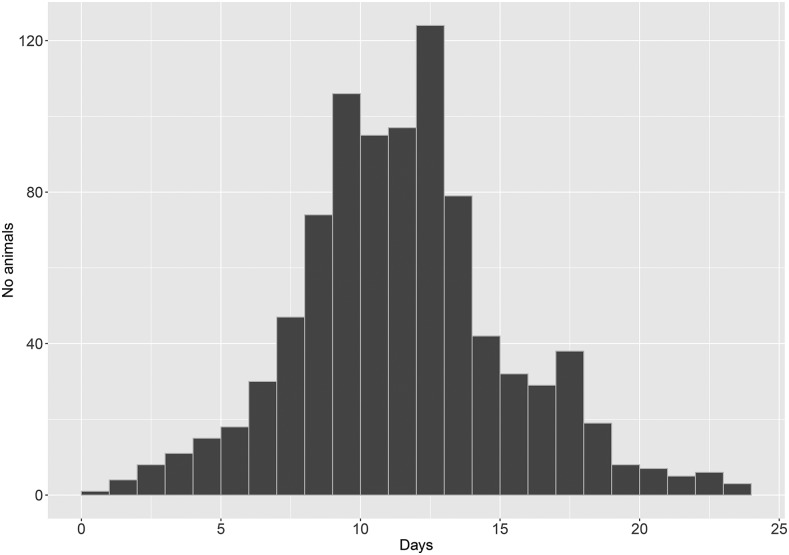
Daily mortality levels of fish during the KHV challenge experiment.

Mortalities began at 12 dpi reaching a maximum between 21 and 24 dpi (98 – 130 mortalities per day) decreasing thereafter with no mortalities observed after 35 dpi (Figure 1). The overall mortality in the KHV challenge experiment for the Amur Mirror Carp was 66%. All observed mortalities displayed typical KHV symptoms (*e.g.*, weakness, lethargy, loss of equilibrium, erratic swimming, sunken eyes, excessive mucous production, increased respiratory rate, discoloration, and hemorrhagic lesions on the skin and gills). The presence of KHV was confirmed in all tested samples (n = 100).

### Heritability estimation

There was marked between-family variation in survival rate for both sires (6–83%) and dams (0–52%), suggesting the existence of considerable genetic variation for host resistance. Heritability estimates of overall survival for the pedigree and genomic relationship matrix on the underlying scale were 0.61 (HPD interval 95%: 0.42 – 0.80) and 0.50 (HPD interval 95%: 0.38 – 0.63) respectively.

### Genome wide association approaches - SNP annotation in QTL region

**Figure 2:**
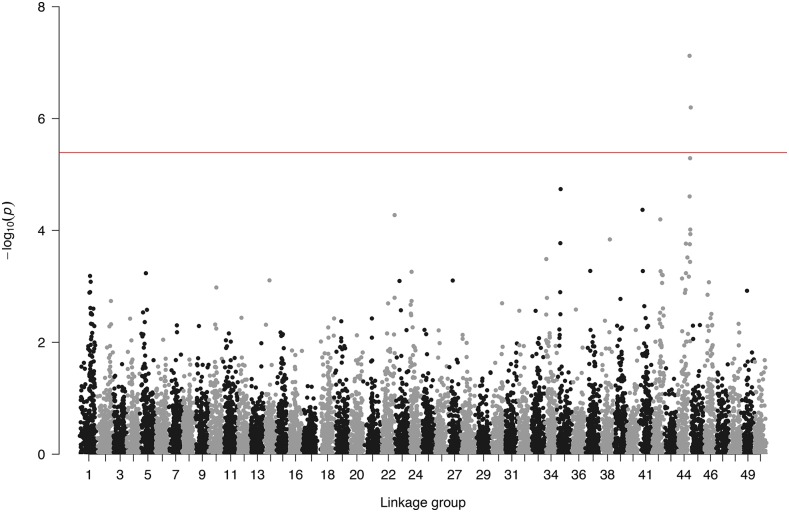
Classical Genome wide association plot for overall survival during the KHV challenge.

**Figure 3:**
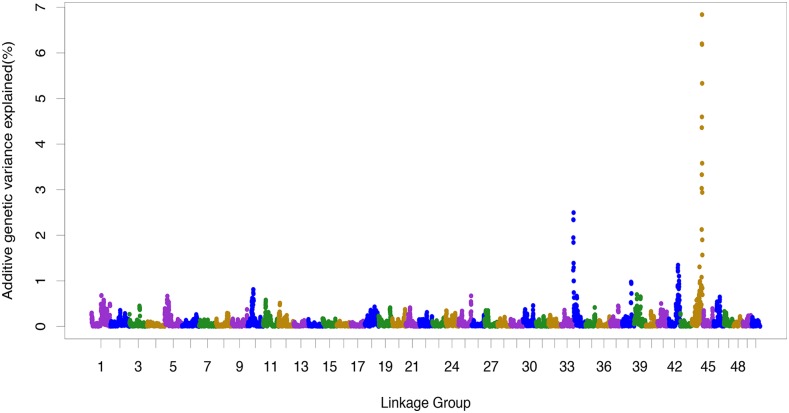
WGBLUP for resistance to KHV. The additive genetic variance explained was calculated using windows of 10 adjacent SNPs.

**Figure 4:**
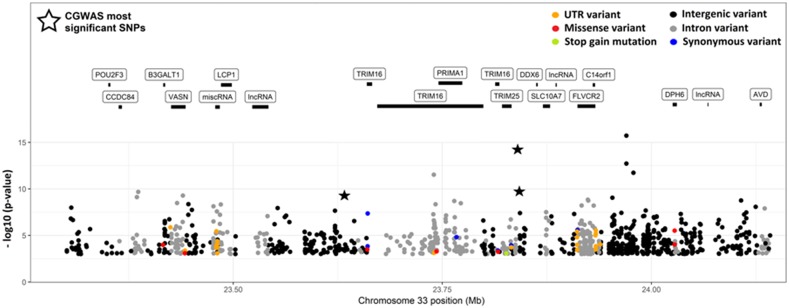
Annotation of the QTL region on chromosome 33 (linkage group 44) including identification of putative genes in the region, functional annotation of SNPs.

The three SNPs with the highest association according to classical GWAS were located on linkage group 44 (chromosome 33; *P* < 1e-05; denoted by stars in Figure 2). This QTL was also identified using the WGBLUP approach (Figure 3) suggesting it accounted for approximately 7% (convergence obtained after 5 iterations) of the additive genetic variance on the underlying scale. In addition the WGBLUP identified QTL explaining more than 1% of the additive genetic variance in linkage groups 34 (∼2.5%) and 42 (∼1.1%). Whole genome sequencing data from the pools of resistance and susceptible animals was used to discover and annotate additional SNPs in the QTL region (Figure 4), and potential candidate genes were identified. Further, SNPs with significant allele frequency differences (P-value < 0.05) between the two groups were identified. A SNP coding for a putative premature stop codon was identified in gene TRIM25 (Glu258*), an E3 ubiquitin ligase with a major role in initiation of intracellular antiviral response to herpesviruses (Gupta *et al.* 2018).

## Discussion

In the current study, high throughput sequencing was applied to study genetic resistance of common carp to KHV. While genomic data in the form of genetic markers can be a valuable addition to selective breeding for disease resistance, how to apply this data depends on the underlying genetic architecture. In the case of traits controlled by a major QTL, it may be most effective to use marker-assisted selection, while in the case of polygenic traits genomic selection is likely to be preferable. Modern genomic tools also facilitate high resolution study of the genomic regions underpinning genetic resistance, facilitating identification and annotation of promising functional candidate genes which may play a direct role in differential host response to infection.

Following pedigree reconstruction using the RAD SNP data, the heritability of resistance as measured by survival on the underlying scale was estimated to be 0.61 (pedigree) and 0.50 (genomic). This is an unusually high heritability estimate, but is comparable to the estimated of 0.79 that was previously documented for this trait (Ødegård *et al.* 2010). These independent high estimates of heritability of resistance to KHV highlight that selective breeding has major potential for producing carp with increased resistance. Additionally, in a recent study of introgression of KHV resistance from a wild carp strain to a farmed carp strain, significant additive genetic variation in resistance was detected (Tadmor-Levi *et al.* 2017). Furthermore, the authors showed that resistant carp do become infected, implying that resistance is due to an effective host response to infection (Tadmor-Levi *et al.* 2017). Early stage host response to KHV infection is likely to have a major interferon pathway component, with Interferon αβ, and interleukin 12 suggested to play a major role in Koi and Red common carp (Hwang *et al.* 2017).

The CGWAS resulted in the identification of genome-wide significant QTL on linkage group 44. While this test is the most commonly used association analysis, it fails to utilize all available information since it does not consider linkage disequilibrium between adjacent SNPs, resulting in reduced statistical power as opposed to methods where all SNPs are used simultaneously (Wang *et al.* 2012). The WGBLUP approach incorporates multiple SNPs and combines the computational efficiency of GBLUP with an increased statistical power for QTL detection (Zhang *et al.* 2016). However, WGBLUP has limitations as well like the heuristic influence regarding optimal number of iterations and the difficulty to determine appropriate significance levels for the identified QTL (Wang *et al.* 2012; Lourenco *et al.* 2015; Zhang *et al.* 2016). The recent implementation of nonlinearA (VanRaden 2008) in PostGSF90 (Misztal *et al.* 2018) may help circumvent the issue of optimal number of iterations due to its better convergence properties. NonlinearA benefits particularly in situations where a non normal prior distribution more accurately describes the trait under study (VanRaden 2008). In the current study, both CGWAS and WGBLUP provided significant evidence for the existence of a QTL associated with resistance to KHV on linkage group 44, explaining approximately 7% of the genetic variation in a highly heritable trait.

The SNP with highest association in the CGWAS was located ∼6.5 Kb upstream of TRIM25, an E3 ubiquitin ligase with a major role in initiation of intracellular antiviral response to herpesviruses. Auto-ubiquitinisation of TRIM25 is a viral strategy for functional inactivation of the pattern recognition protein RIG1, and subsequent cellular interferon response (Gack *et al.* 2008). In the PWGS, the majority of the SNPs with significant allele frequency differences between the resistant and susceptible pools were annotated as ‘intergenic’. However, interestingly, a putative premature stop mutation in position 258 of the carp TRIM25 protein was identified. TRIM25 has 649 - 682 amino acids (isoform dependant), and therefore this stop mutation is highly likely to result in loss of function. The premature stop causing allele is rare in the population, but reads of this allele were more common in the susceptible (n = 11) than the resistance (n = 3) pools, albeit the Cochran-Mantel-Haenszel test p-value for this SNP was only nominaly significant (0.049). This may fit with a loss of function of TRIM25 in susceptible fish, being unable to trigger an appropriate antiviral response.

It will be interesting to study whether this single genome-wide significant QTL for resistance to KHV has an effect in other carp populations and strains. Follow up functional studies of candidate genes in the QTL region, including assessment of gene expression response to infection and the differential response between alternate QTL types, may be a fruitful avenue to shortlist functional candidate genes. Currently, TRIM25 and its premature stop mutation seem to be the most promising candidates, and additional genotyping of this SNP alongside directed functional studies may help to test if it may be causative for the QTL. While the QTL identified in the current study was highly significant, the proportion of genetic variation explained was relatively moderate, implying multifactorial causal mechanisms underlying host resistance. Nonetheless, it is plausible that genetic markers within the QTL region may have value for marker-assisted selection, either directly or via a genomic prediction strategy with increased weighting on QTL-region SNPs.

### Conclusions

In conclusion, the results from the current study demonstrate that SNP markers generated via RADseq are effective at studying the genetic variation in resistance to KHV in a common carp breeding population. The RAD-derived SNPs facilitated the identification of a genome-wide significant QTL on LG 44 (chromosome 33) affecting resistance to KHV. The sequencing and annotation of the QTL regions provided candidate functional genes and polymorphisms for future study to understand the mechanisms underlying the QTL. This QTL may have value for selective breeding via incorporation into marker-assisted or genomic selection, albeit genetic resistance to KHV in common carp appears to be multifactorial in nature.
